# Supplementary figures and images for: YTHDC1 m6A-dependent and m6A-independent functions converge to preserve the DNA damage response
Source: EMBO J. 2024 Jul 1;43(16):10. doi: 10.1038/s44318-024-00153-x (PMC11329685; doi:10.1038/s44318-024-00153-x)

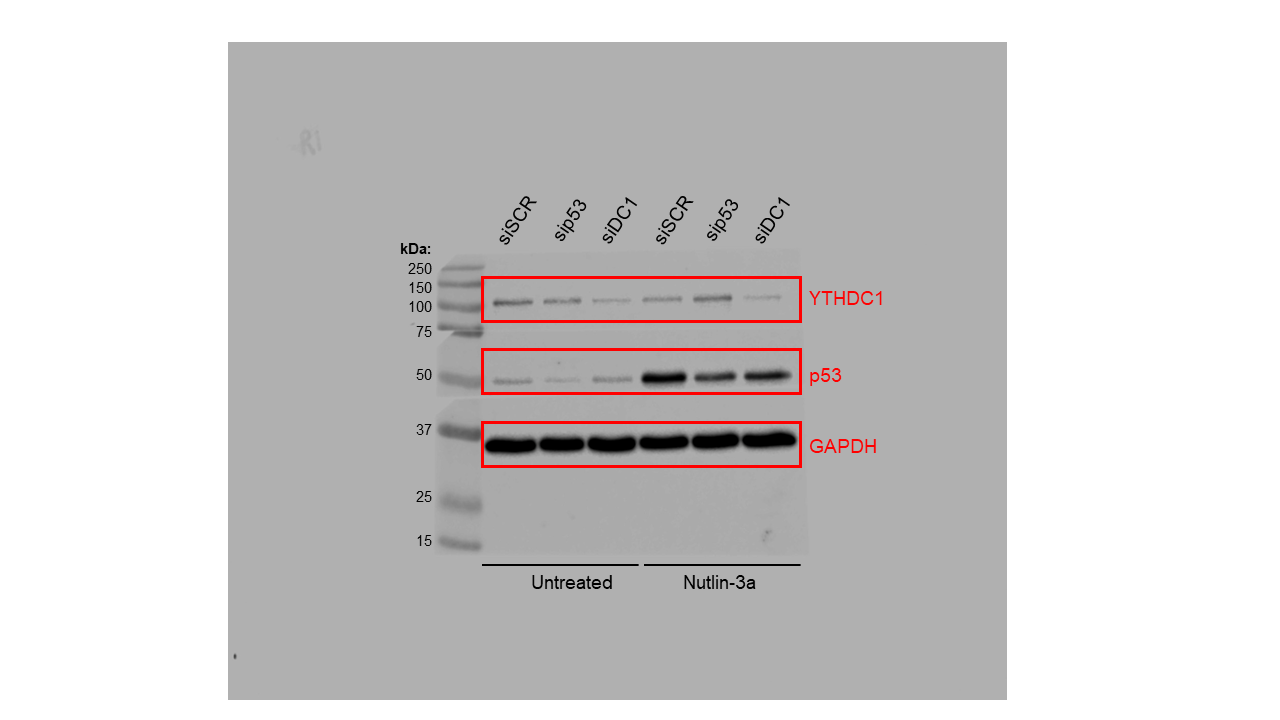

Supplement: Supplementary file 7 — Source data Fig. 2 [file 44318_2024_153_MOESM7_ESM.zip › Figure 2/2A/Picture 2A.tif]

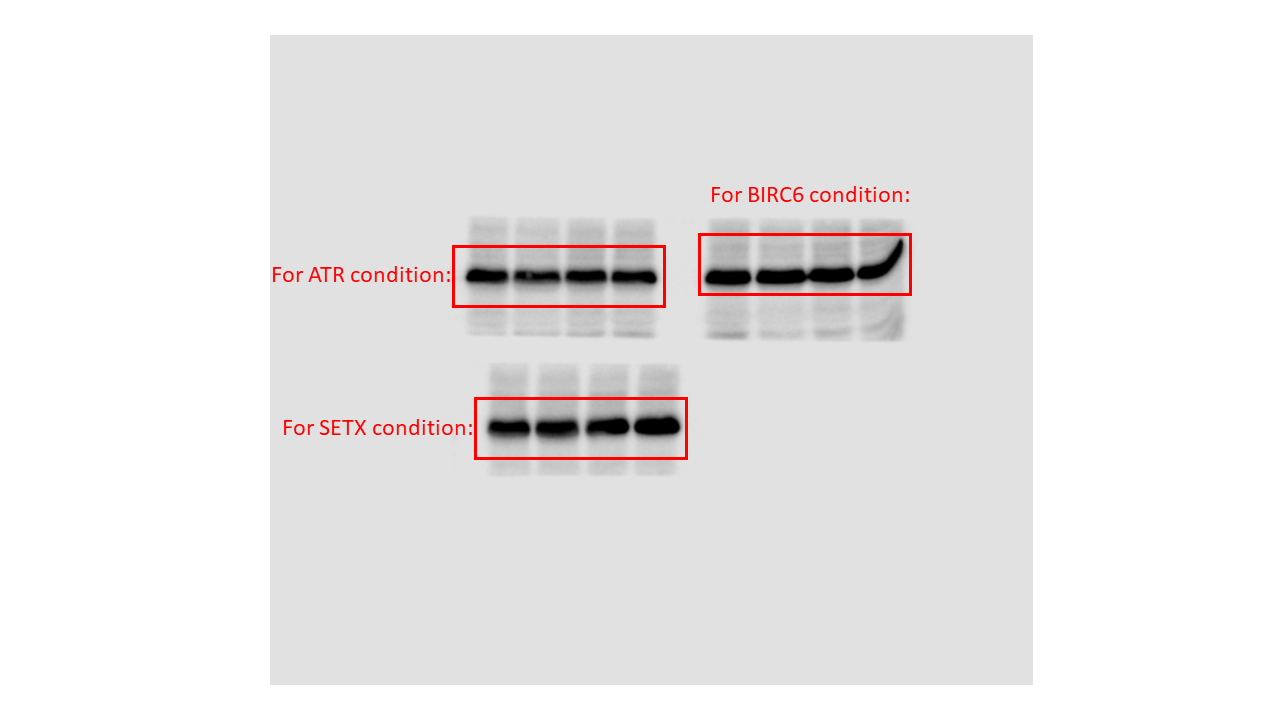

Supplement: Supplementary file 9 — Source data Fig. 4 [file 44318_2024_153_MOESM9_ESM.zip › Figure 4/4D-F/Alfa Tubulin (4D-F).tif]

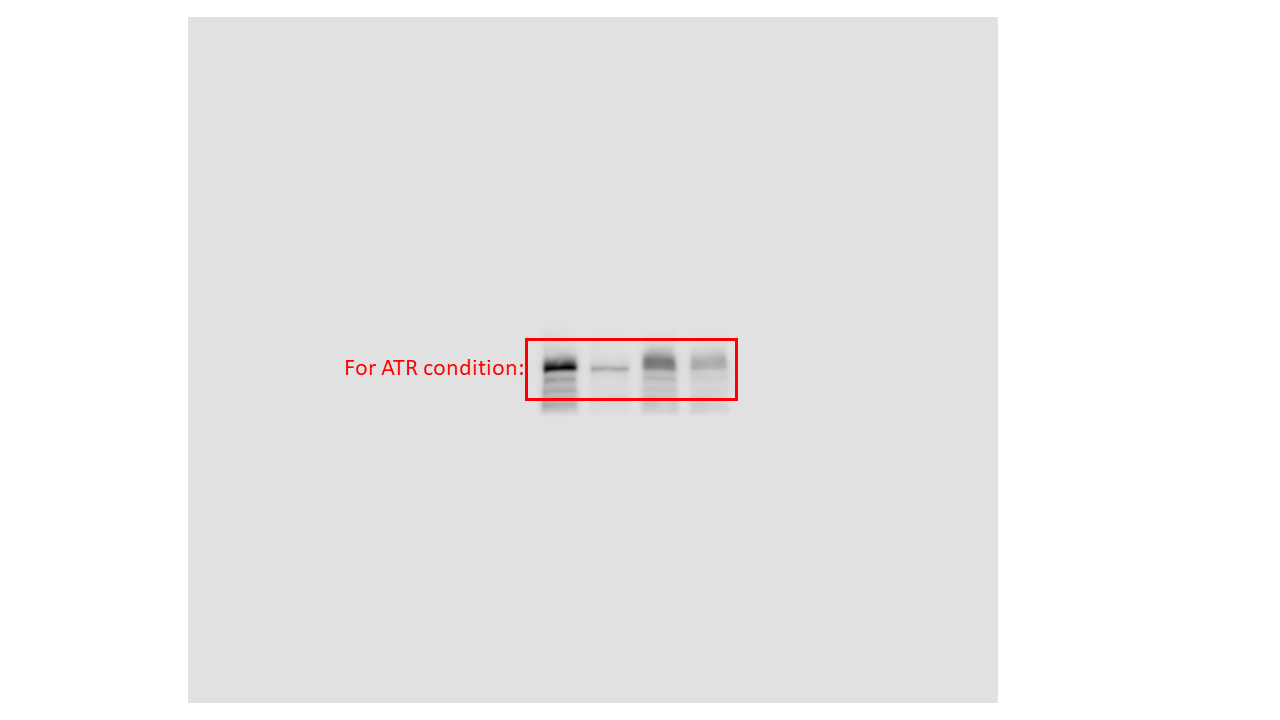

Supplement: Supplementary file 9 — Source data Fig. 4 [file 44318_2024_153_MOESM9_ESM.zip › Figure 4/4D-F/ATR (4D).tif]

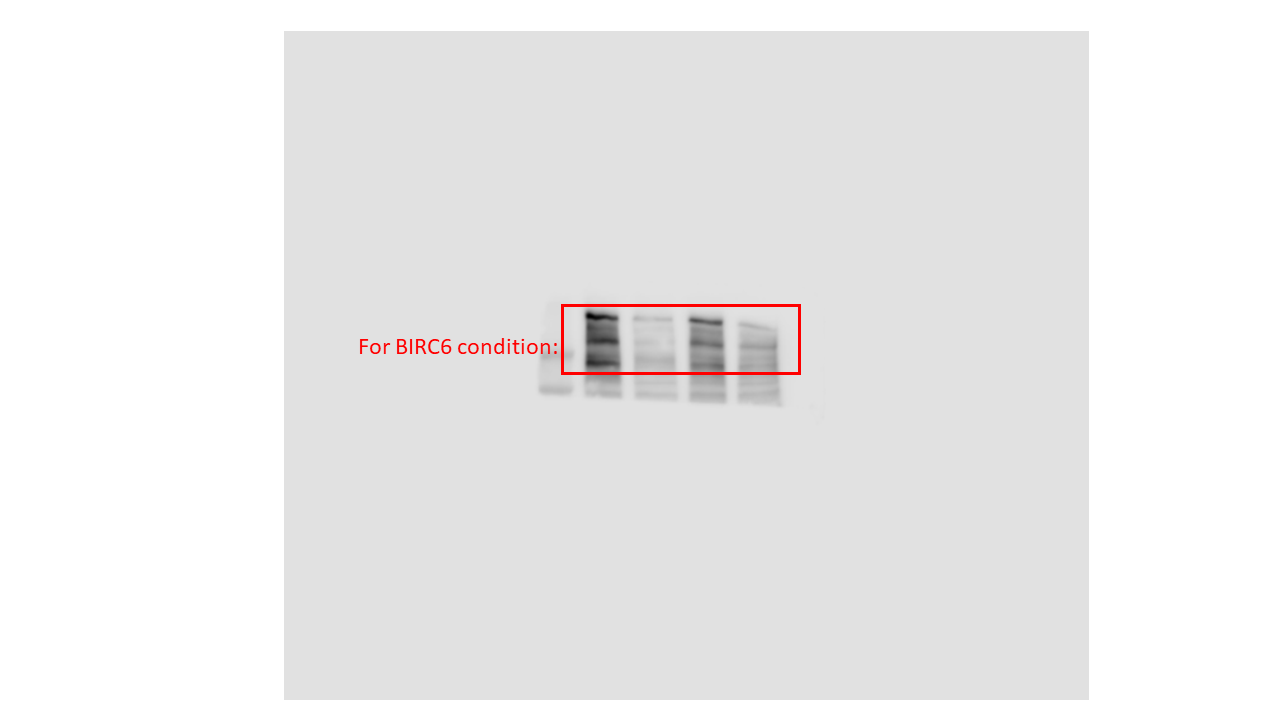

Supplement: Supplementary file 9 — Source data Fig. 4 [file 44318_2024_153_MOESM9_ESM.zip › Figure 4/4D-F/BIRC6 (4E).tif]

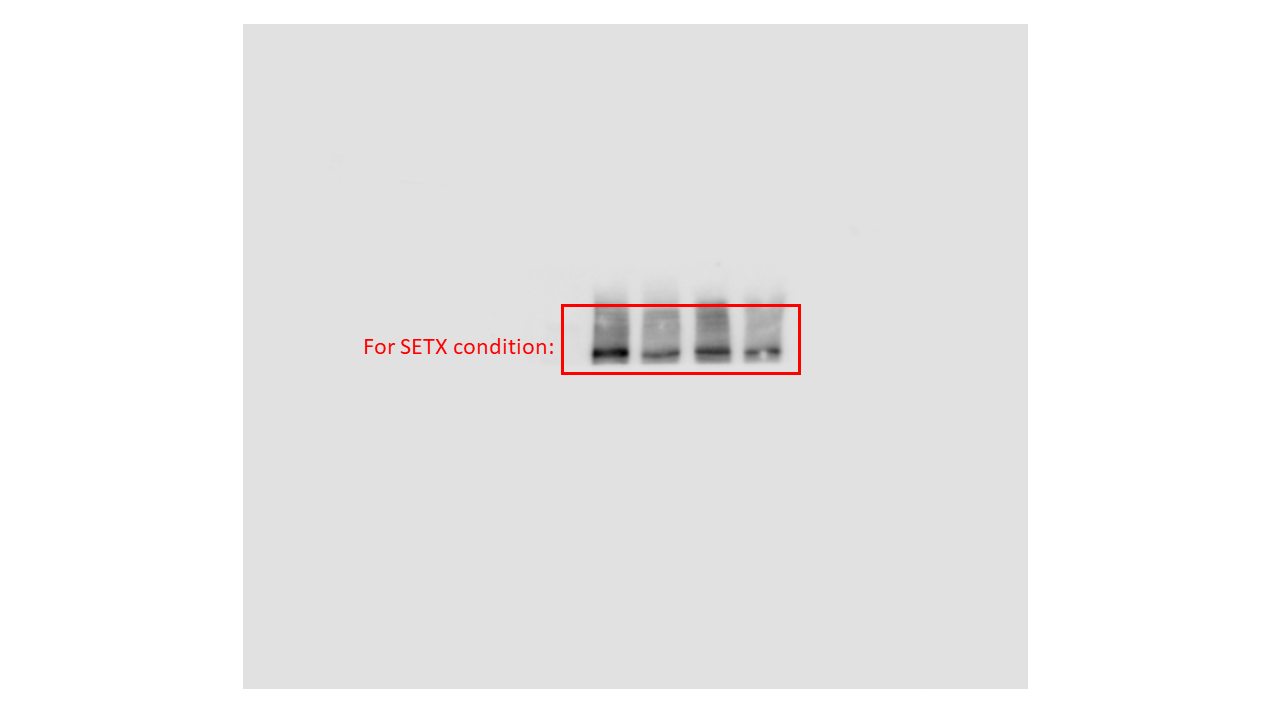

Supplement: Supplementary file 9 — Source data Fig. 4 [file 44318_2024_153_MOESM9_ESM.zip › Figure 4/4D-F/SETX (4F).tif]

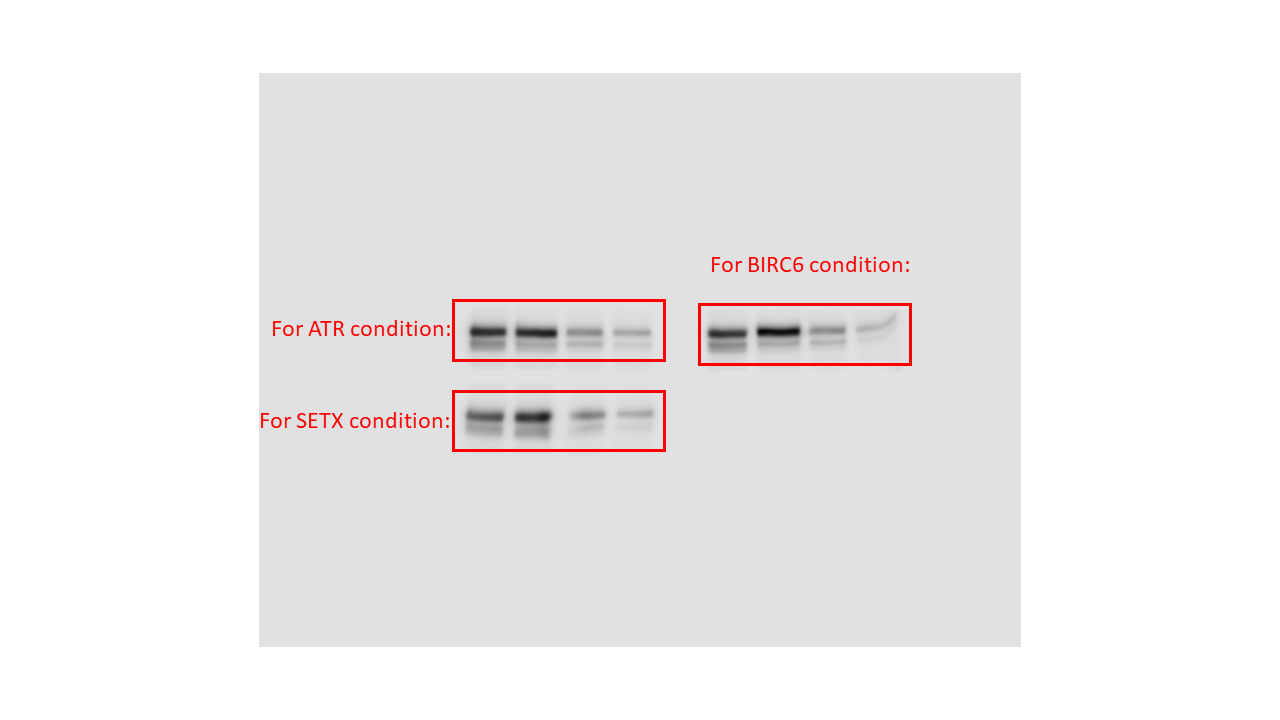

Supplement: Supplementary file 9 — Source data Fig. 4 [file 44318_2024_153_MOESM9_ESM.zip › Figure 4/4D-F/YTHDC1 (4D-F).tif]

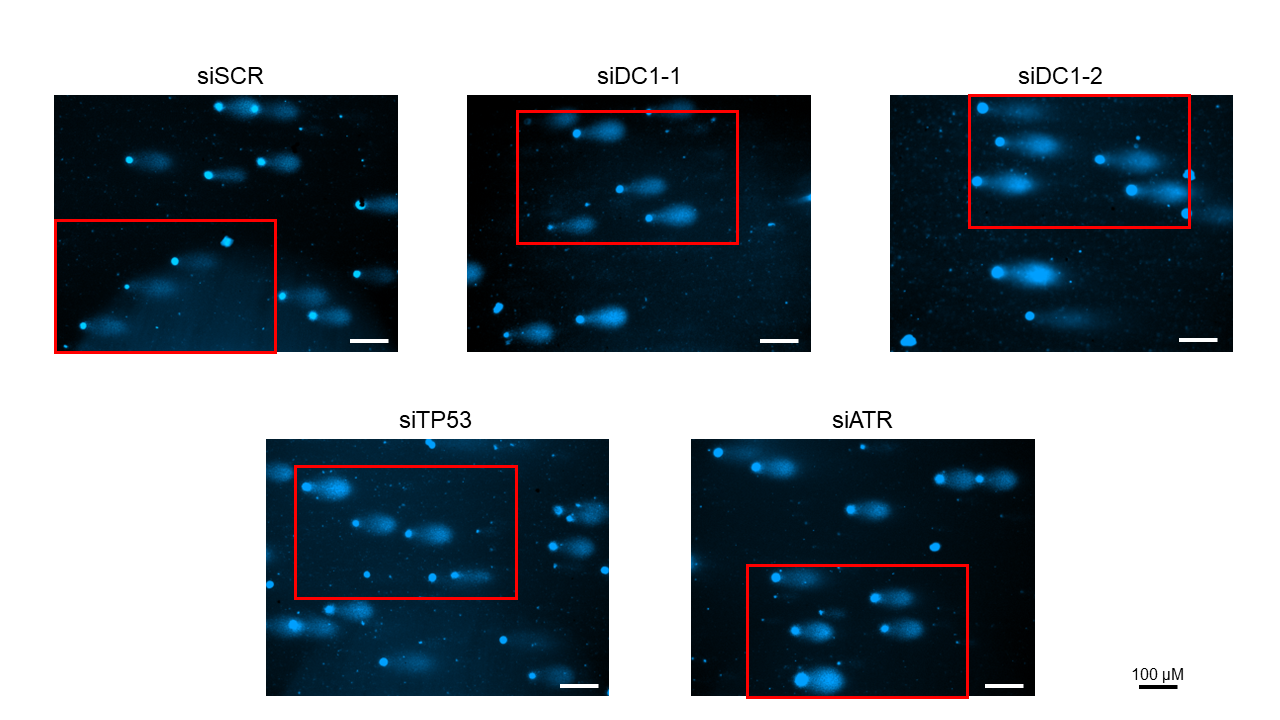

Supplement: Supplementary file 11 — Source data Fig. 6 [file 44318_2024_153_MOESM11_ESM.zip › Figure 6/6A-B/Picture 6A.tif]

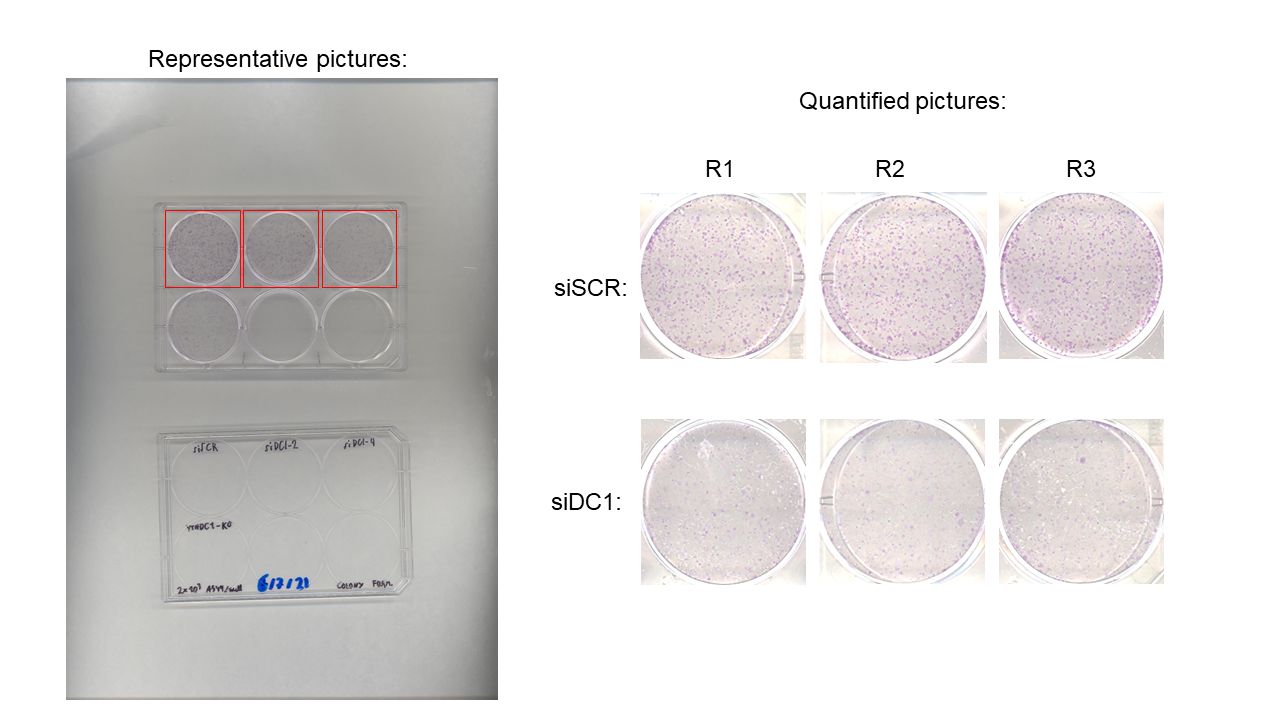

Supplement: Supplementary file 11 — Source data Fig. 6 [file 44318_2024_153_MOESM11_ESM.zip › Figure 6/6F/Pictures 6F.tif]

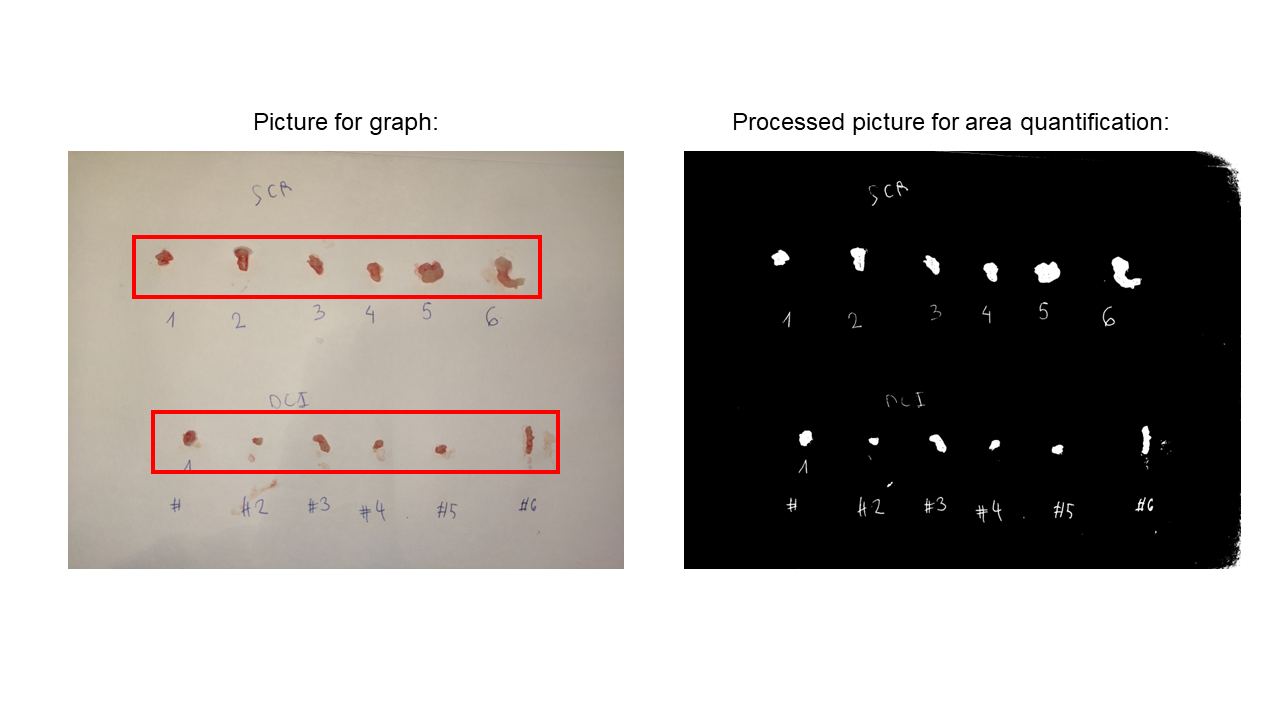

Supplement: Supplementary file 11 — Source data Fig. 6 [file 44318_2024_153_MOESM11_ESM.zip › Figure 6/6G/Picture 6G.tif]
