# Supplementary material for: YTHDC1 m6A-dependent and m6A-independent functions converge to preserve the DNA damage response
Source: EMBO J. 2024 Jul 1;43(16):10. doi: 10.1038/s44318-024-00153-x (PMC11329685; doi:10.1038/s44318-024-00153-x)
Supplement: Supplementary file 12 — Expanded View Figures [file 44318_2024_153_MOESM12_ESM.pdf]

Expanded View Figures

Figure EV1. CRISPR screening enables unbiased identification of RNA modifiers involved in p53 response.

(**A**) Representative Immunoblot of p53, with GAPDH as loading control. Cells were treated with Nutlin-3a or untreated as negative control. (**B**) Nutlin-3a dose response treatment. P21-Reporter cells were treated with increasing doses of Nutlin-3a or untreated as negative control. After treatment, reporter gene activation was measured by flow cytometry. The percentage of cells showing reporter gene activation was calculated and presented in the plot of the signal distribution. (**C**) Representative immunoblot of spCas9, with a-tubulin as loading control. Cells were transduced with a lentivirus carrying spCas9 or an empty vector as negative control. (**D**) Flow cytometry quantification of P21-Reporter gene activation and CRISPR library infection for the two replicates used in the screening. (**E**) Scatter plot of log_2 fold change for sgRNA enrichment in p53-enhanced and p53-attenuated populations. Each individual sgRNA for the three top candidates for each population is labelled and highlighted in the plot. Non-targeting sgRNAs used as negative controls in black. (**F**) Scatter plot of log_2 fold change for gene enrichment in p53-enhanced and p53-attenuated populations. Previously reported negative or positive regulators of p53 activity are labelled in the plot in red and green, respectively. (**G**) Functional annotation of the top 50 candidates for p53-enhanced and p53-attenuated populations based on their molecular substrate. (**H**) RNA-level quantification of mature mRNA by RT-qPCR for *YTHDC1* and *ASH2L*. Cells were transfected with two independent siRNA against *YTHDC1* (DC1-1 and -2), two independent siRNA against *ASH2L* (ASH2L-1 and -2) or Scramble (SCR) as negative control. Data information: All data are shown are representative of at least three independent experiments, except for (**C**). Data are presented as mean ± s.d. ***$P \leq 0.001$, ****$P \leq 0.0001$, paired two-tailed Student's *t* test was performed in (**H**).

 © The Author(s)

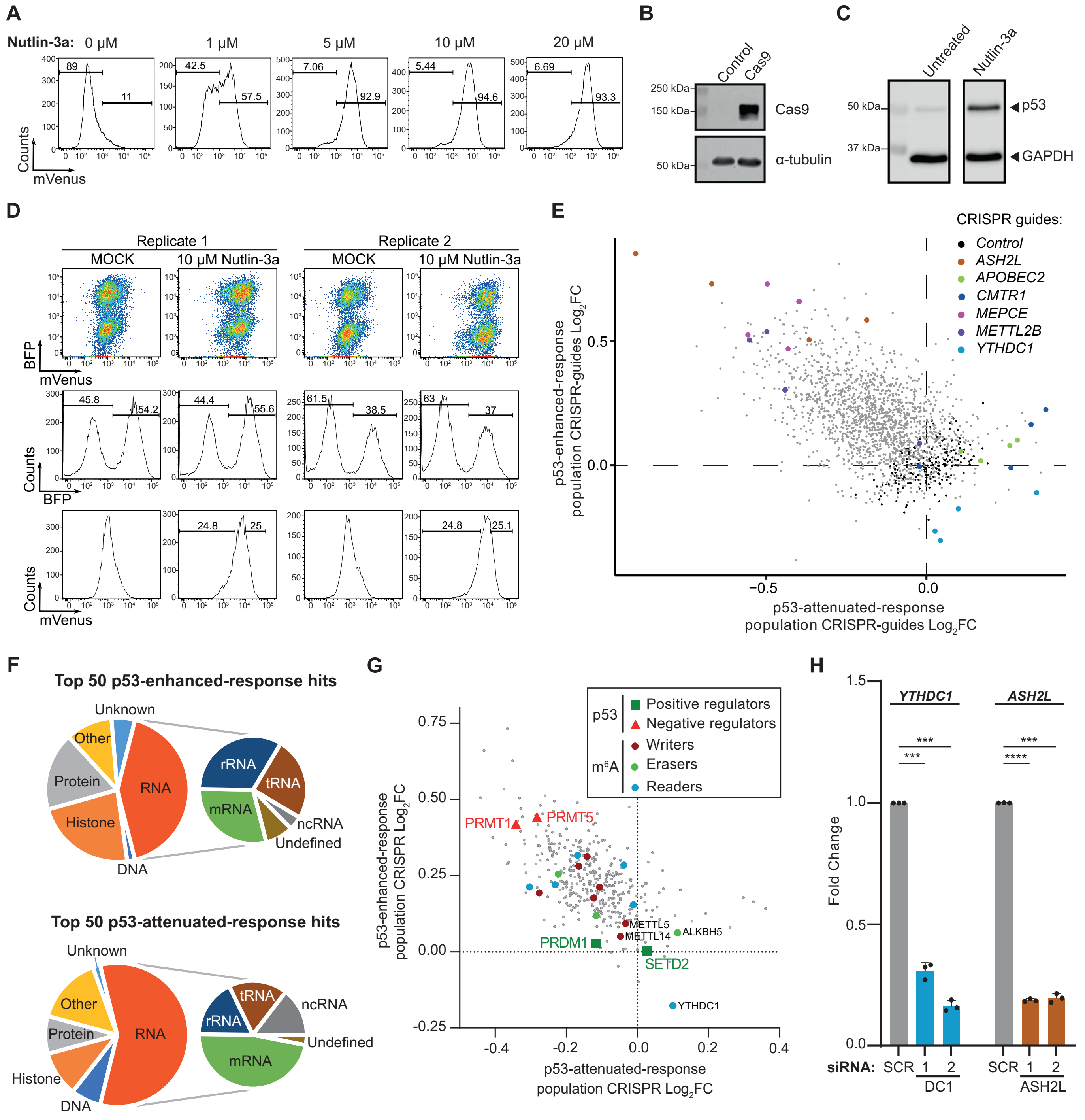
A
Nutlin-3a: 0 µM
1 µM
5 µM
10 µM
20 µM
89
11
42.5
57.5
7.06
92.9
5.44
94.6
6.69
93.3
Counts
mVenus
B
Control
Cas9
250 kDa
150 kDa
50 kDa
Cas9
α-tubulin
C
Untreated
Nutlin-3a
50 kDa
37 kDa
p53
GAPDH
D
Replicate 1
Replicate 2
MOCK
10 µM Nutlin-3a
BFP
mVenus
45.8
54.2
44.4
55.6
61.5
38.5
63
37
Counts
BFP
24.8
25
24.8
25.1
Counts
mVenus
E
CRISPR guides:
Control
ASH2L
APOBEC2
CMTR1
MEPCE
METTL2B
YTHDC1
p53-enhanced-response population CRISPR-guides Log2FC
p53-attenuated-response population CRISPR-guides Log2FC
F
Top 50 p53-enhanced-response hits
Unknown
Other
Protein
RNA
Histone
DNA
rRNA
tRNA
mRNA
ncRNA
Undefined
Top 50 p53-attenuated-response hits
G
p53
Positive regulators
Negative regulators
m6A
Writers
Erasers
Readers
PRMT1
PRMT5
METTL5
METTL14
ALKBH5
PRDM1
SETD2
YTHDC1
p53-enhanced-response population CRISPR Log2FC
p53-attenuated-response population CRISPR Log2FC
H
YTHDC1
ASH2L
***
****
Fold Change
siRNA: SCR
DC1
ASH2L

© The Author(s)

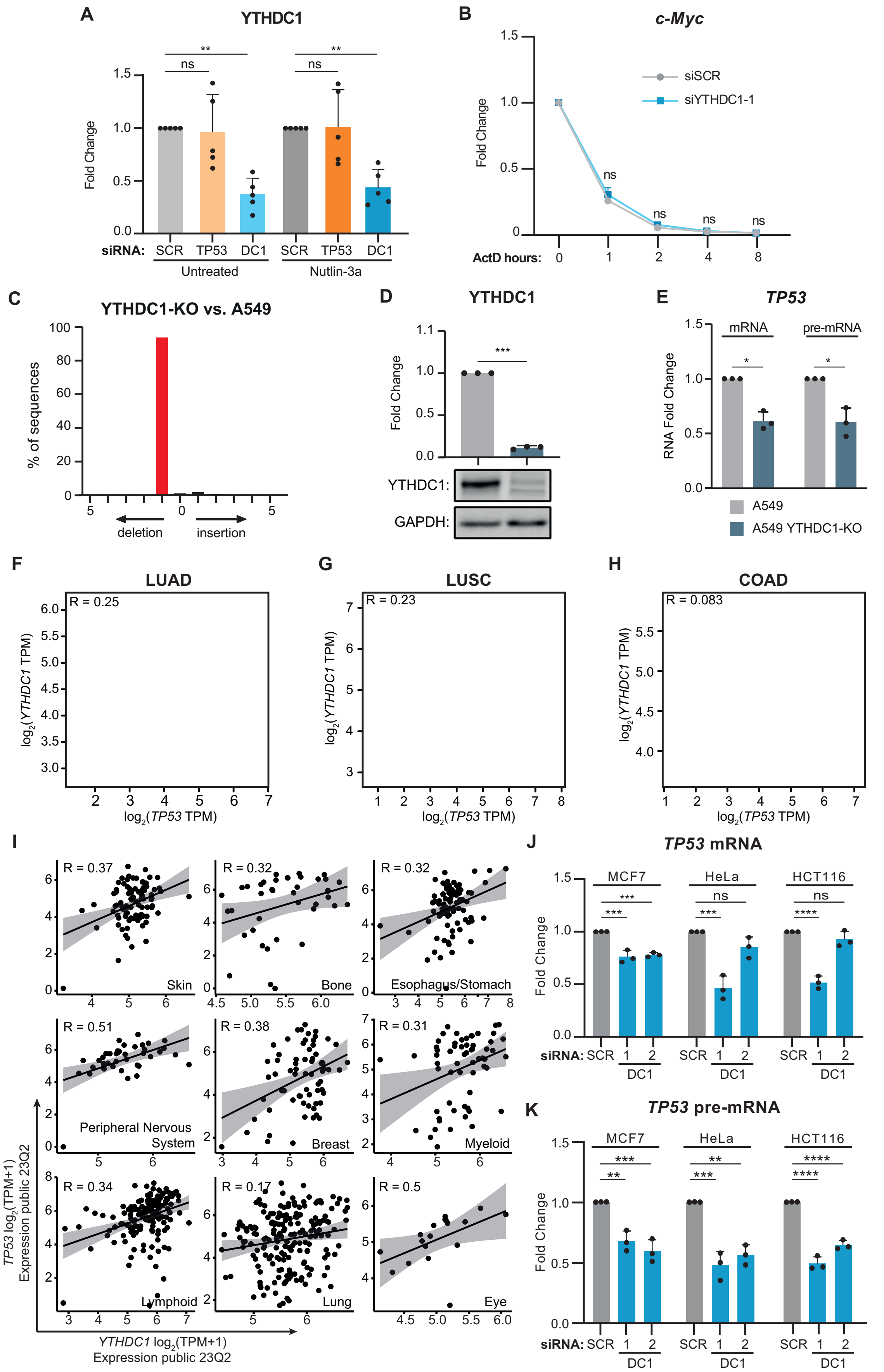
A
YTHDC1
Fold Change
siRNA: SCR TP53 DC1 SCR TP53 DC1
Untreated
Nutlin-3a
B
c-Myc
siSCR
siYTHDC1-1
Fold Change
ActD hours: 0 1 2 4 8
C
YTHDC1-KO vs. A549
% of sequences
deletion
insertion
D
YTHDC1
Fold Change
YTHDC1:
GAPDH:
E
TP53
mRNA
pre-mRNA
RNA Fold Change
A549
A549 YTHDC1-KO
F
LUAD
R = 0.25
G
LUSC
R = 0.23
H
COAD
R = 0.083
I
Skin
Bone
Esophagus/Stomach
Peripheral Nervous System
Breast
Myeloid
Lymphoid
Lung
Eye
TP53 log2(TPM+1) Expression public 23Q2
YTHDC1 log2(TPM+1) Expression public 23Q2
J
TP53 mRNA
MCF7
HeLa
HCT116
K
TP53 pre-mRNA
siRNA: SCR 1 2
DC1

 © The Author(s)

Figure EV2. YTHDC1 directly regulated TP53 transcription.

(**A**) YTHDC1 protein quantification. Cells were transfected with siRNA against *TP53*, *YTHDC1* or Scramble (SCR) as negative control. After silencing cells were treated to Nutlin-3a, DMSO was used for the untreated condition as negative control. The bar plot shows protein quantification relative to GAPDH level of $n = 5$ biologically independent experiments. (**B**) *c-Myc* RNA stability assay. Cells were transfected with siRNA, YTHDC1, or Scramble (SCR) as negative control. After silencing cells were treated with Actinomycin D to stop the transcription. Cells were collected at different time points indicated on the X axis to assess *c-Myc* RNA level by quantitative RT-qPCR. In vitro transcribed *Luciferase* RNA was used as spike-in to normalise the signal. (**C**) Comprehensive profile of insertions and deletions (indels) in YTHDC1-KO A549 clone compared to a control A549 cells. (**D**) Representative picture of a Western blot of normal A549 cell line and the YTHDC1-KO A549 clone, together with relative quantification of 3 independent protein extractions from the same clone. (**E**) RNA-level quantification of mature mRNA and pre-mRNA by RT-qPCR for *TP53* in A549 cells (grey) and YTHDC1-KO (dark blue). (**F–H**) Correlation plot showing *YTHDC1* and *TP53* expression (as log2 TPM + 1 pseudocounts) in LUAD samples ($n = 505$) (**B**), in LUSC samples ($n = 479$) (**C**), and COAD samples ($n = 427$) (**D**) from TCGA database (gdc-portal.nci.nih.gov). Correlation *P* value is calculated using a t-distribution. (**I**) Correlation plot showing *YTHDC1* and *TP53* expression (as log2 TPM + 1 pseudocounts) in 824 cell lines grouped based on the tissue of origin from DepMap database (Tsherniak et al, 2017, https://depmap.org/portal). Correlation *P* value is calculated using a t-distribution. (**J**) RNA-level quantification of mature mRNA by RT-qPCR for *TP53* in multiple cell lines. MCF7, HeLa and HCT116 cell lines were transfected with two independent siRNA against *YTHDC1* (DC1-1 and -2), or Scramble (SCR) as negative control. (**K**) RNA-level quantification of pre-mRNA by RT-qPCR for *TP53* in multiple cell lines. MCF7, HeLa and HCT116 cell lines were transfected with two independent siRNA against *YTHDC1* (DC1-1 and -2), or Scramble (SCR) as negative control. Data information: All data are shown are representative of at least three independent experiments. Data are presented as mean ± s.d. ns, not significant $P > 0.05$, *$P \le 0.05$, **$P \le 0.01$, ***$P \le 0.001$, ****$P \le 0.0001$, paired two-tailed Student's *t* test was performed in (**A**, **B**, **D**, **E**, **J**, **K**).

© The Author(s)

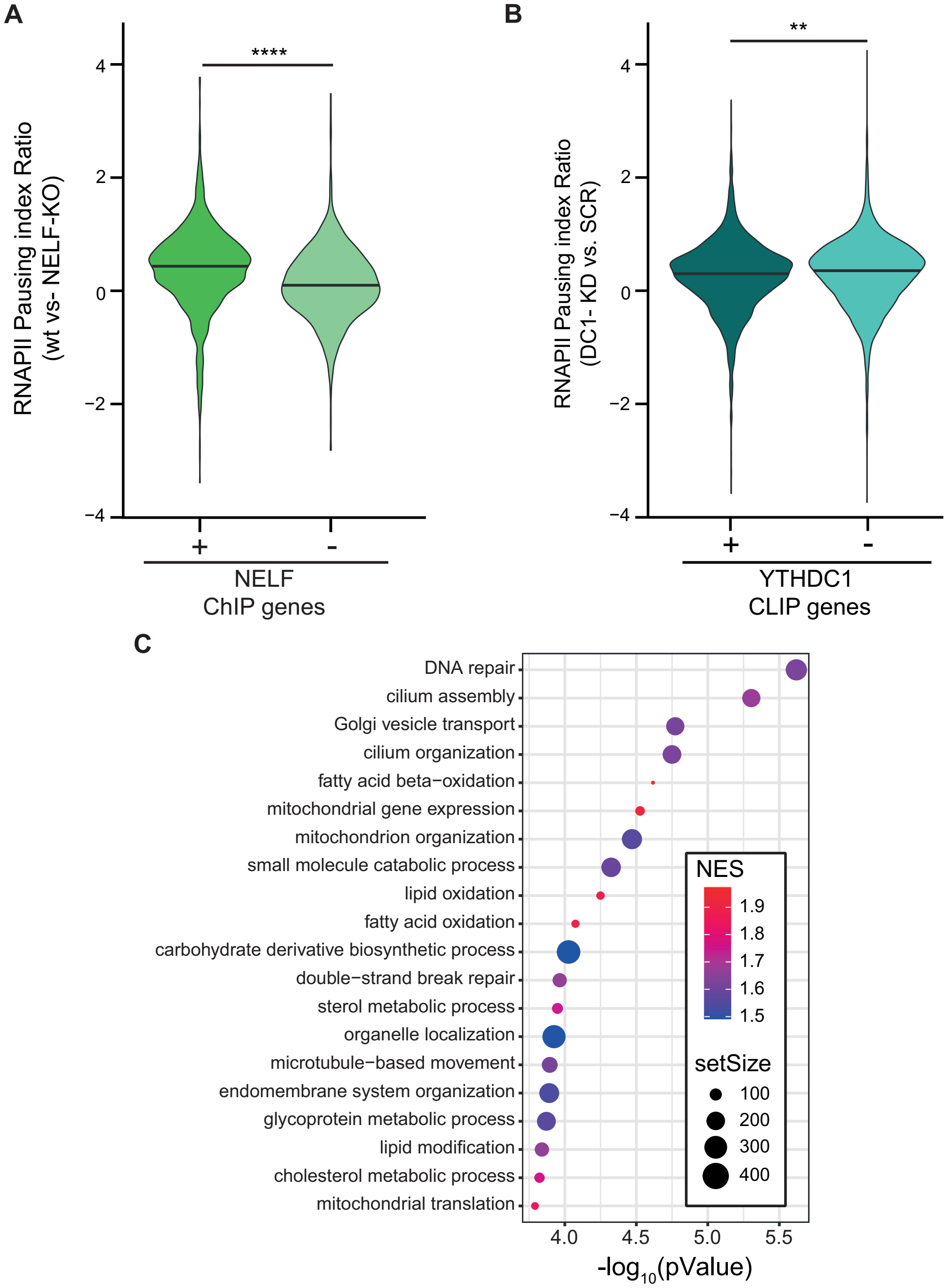

Figure EV3. RNAPII pausing upon YTHDC1 depletion mainly affects DNA repair.

(**A**) Violin plots of the distribution of expression corrected RNAPII Pausing Index ratio calculated for public dataset NELF ChIP-seq experiment. All the actively transcribed genes were divided into two different groups based on the presence or absence of NELF peaks in the TSS (green and light green blue, respectively). Pausing index was calculated as log2 RNAPII promoter density/RNAPII gene body density for both wild-type and NELK-KO cells. Data are presented as distribution of Pausing Index ratio for wild-type versus KO. (**B**) Violin plots of the distribution of expression corrected RNAPII Pausing Index ratio calculated for YTHDC1-CLIP-seq dataset. All the actively transcribed genes were divided into two different groups based on the presence or absence of YTHDC1 along the transcript (dark and light blue bondi). Pausing index was calculated as log2 RNAPII promoter density/RNAPII gene body density for both cells transfected with scramble (SCR) siRNA and YTHDC1 siRNA. Data are presented as distribution of Pausing Index ratio for YTHDC1 knockdown versus SCR. (**C**) Gene Set Enrichment Analysis (GSEA) of genes with an increased RNAPII Pausing Index upon YTHDC1 depletion. Data information: All data are shown are representative of at least three independent experiments. Data are presented as mean ± s.d. $^{**}P \leq 0.001$, $^{****}P \leq 0.00001$, paired two-tailed Student's *t* test was performed in (**A**, **B**). GSEA has been performed following Subramanian algorithm (Subramanian et al, 2005).

 © The Author(s)

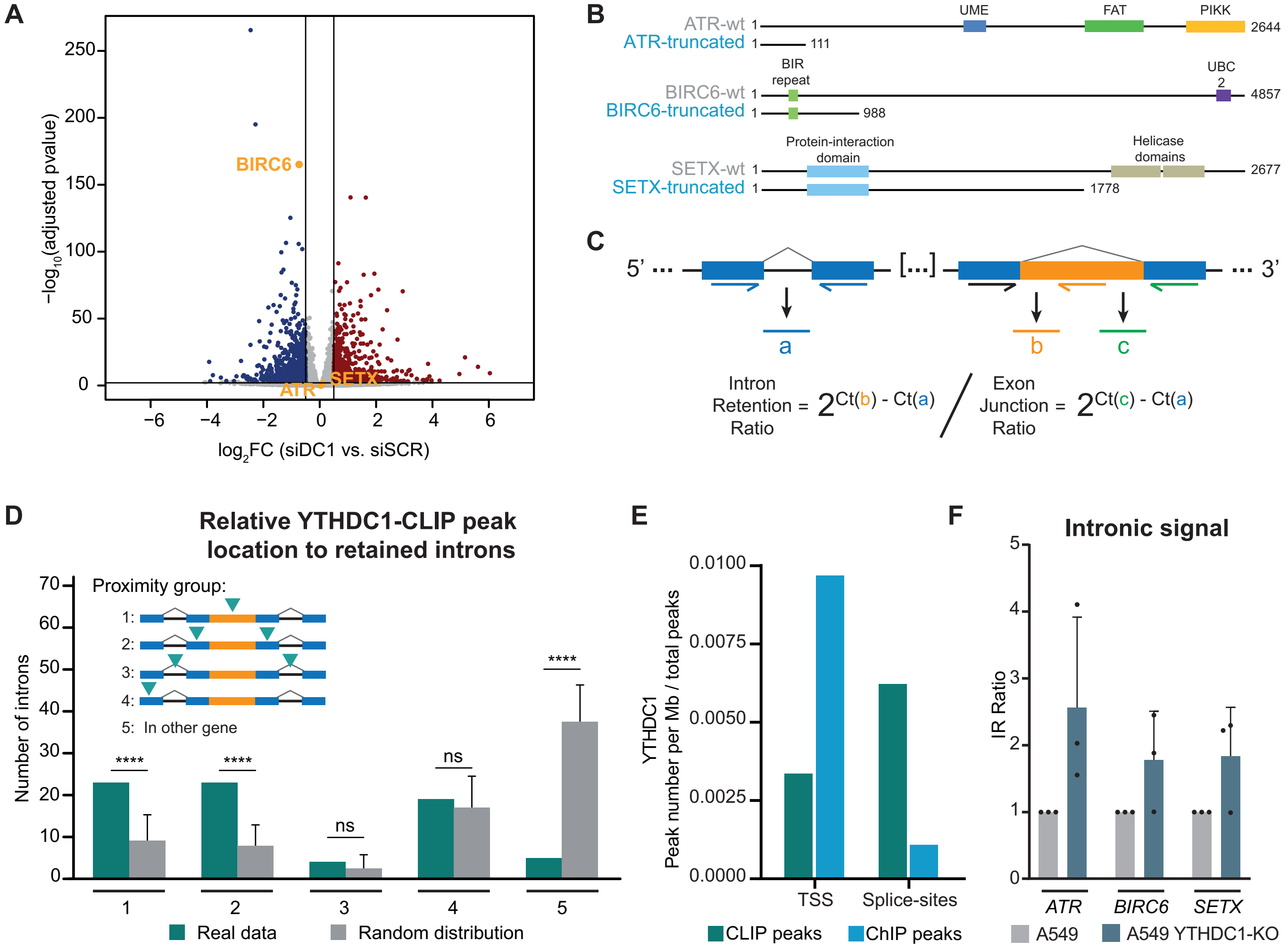

Figure EV4. YTHDC1 directly promotes correct splicing.

(**A**) Volcano plot showing differentially expressed genes (DEGs) identified in YTHDC1-silenced cells versus scramble (SCR) cells. Genes with $\log_2(FC) > 0.5$ and Adjusted *P* value < 0.001, resulting from the DESeq2 DGA, are considered DEGs. Significantly upregulated, downregulated or not changed genes in the YTHDC1 knocked-down cells are labelled in blue, red or grey colour, respectively. (**B**) Schematic representation of truncated-version proteins for ATR, BIRC6 and SETX in negative control condition (grey) and YTHDC1-depleted condition (cyan), due to the emergence of premature stop-codons. (**C**) Schematic representation of primer design for quantitative PCR to detect spliced and unspliced isoforms of *ATR, BIRC6* and *SETX*. An upstream exon-junction region we selected to normalise signal. (**D**) Plot showing the number of differentially retained introns (blue bondi bars) and 1000 random selections of GENCODE v19 introns (grey bars), classified in 5 different groups depending on the proximity to a YTHDC1-CLIP peak (group 1: peak inside the intron; group 2: peak in adjacent exons; group 3: peak in adjacent introns; group 4: peak anywhere inside the gene; group 5: no peak inside the gene). Error bars represent twice the standard deviation. (**E**) Relative distribution of YTHDC1-CLIP (blue bondi) and YTHDC1 ChIP (cyan) peaks over TSS (defined from TSS to 500 bp downstream) and splicing sites (5′ and 3′ splicing sites) along genome. (**F**) RNA-level quantification of intronic retention ratio by RT-qPCR for *ATR, BIRC6* and *SETX* mRNA in A549 cells (grey) and YTHDC1-KO (dark blue). Data information: DESeq2 Wald test *P* value was calculated in (**A**). Empirical *P* value for (**D**) was obtained by randomising 100 times the selected introns to compared. Data are presented as mean ± s.d. ns, not significant $P > 0.05$, ****$P \leq 0.0001$, paired two-tailed Student's *t* test was performed in (**D**, **F**).

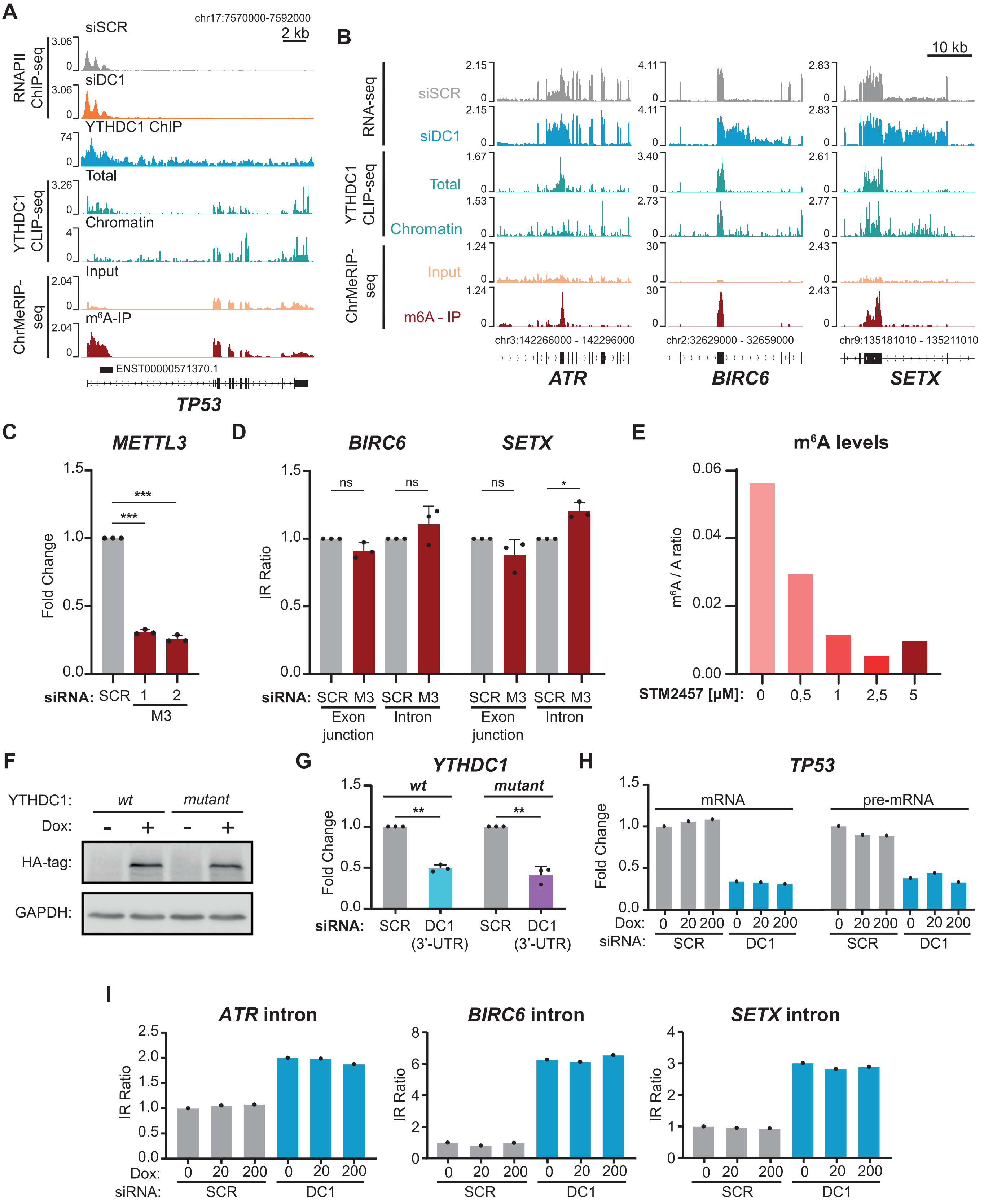
A
chr17:7570000-7592000
2 kb
RNAPII ChIP-seq
siSCR
siDC1
YTHDC1 ChIP
YTHDC1 CLIP-seq
Total
Chromatin
ChrMeRIP-seq
Input
m6A-IP
ENST00000571370.1
TP53
B
10 kb
RNA-seq
siSCR
siDC1
YTHDC1 CLIP-seq
Total
Chromatin
ChrMeRIP-seq
Input
m6A - IP
chr3:142266000 - 142296000
chr2:32629000 - 32659000
chr9:135181010 - 135211010
ATR
BIRC6
SETX
C
METTL3
Fold Change
siRNA: SCR 1 2
M3
D
BIRC6
SETX
IR Ratio
siRNA: SCR M3 SCR M3 SCR M3 SCR M3
Exon junction
Intron
E
m6A levels
m6A / A ratio
STM2457 [µM]: 0 0,5 1 2,5 5
F
YTHDC1: wt mutant
Dox: - + - +
HA-tag:
GAPDH:
G
YTHDC1
wt
mutant
Fold Change
siRNA: SCR DC1 (3'-UTR) SCR DC1 (3'-UTR)
H
TP53
mRNA
pre-mRNA
Fold Change
Dox: 0 20 200 0 20 200
siRNA: SCR DC1
I
ATR intron
BIRC6 intron
SETX intron
IR Ratio
Dox: 0 20 200 0 20 200
siRNA: SCR DC1

 © The Author(s)

Figure EV5. YTHDC1 modulates *TP53* transcription independently of m^{6}A and regulates the correct splicing of *ATR, BIRC6* and *SETX* intron in a m^{6}A-dependent manner.

(**A**) Genome browser tracks for RNAPII ChIP-seq of cell transfected with scramble siRNA (light grey) or siRNA against YTHDC1 (orange), YTHDC1 ChIP (cyan) YTHDC1-CLIP (blue bondi) and ChrMeRIP input (yellow) and m^{6}A specific peaks (dark red), showing reads coverage over *TP53* locus. Sequencing data were normalised as Fragments Per Kilobase of transcript per Million mapped reads (FPKM). (**B**) Genome browser tracks for total RNA-seq of cell transfected with scramble siRNA (light grey) or siRNA against YTHDC1 (cyan), YTHDC1-CLIP (blue bondi) and ChrMeRIP input (yellow) and m^{6}A specific peaks (dark red), showing reads coverage over *ATR*, *BIRC6* and *SETX* retained introns. Sequencing data were normalised as Fragments Per Kilobase of transcript per Million mapped reads (FPKM). (**C**) RNA-level quantification of mature mRNA by RT-qPCR for *METTL3*. Cells were transfected with two independent siRNA against *METTL3* (M3-1 and -2) or Scramble (SCR) as negative control. (**D**) RNA-level quantification of spliced or unspliced for *BIRC6* and *SETX* mRNA by RT-qPCR. Cells were transfected with siRNA against *METTL3* (dark red), or Scramble (light grey) as negative control. (**E**) TLC quantification of the ratio between total amount of Adenosine nucleotides (**A**) and N6-methyladenosine nucleotides (m^{6}A) of one representative replicate. (**F**) Representative western blot of HA-YTHDC1 *wt* and *mutant* versions upon doxycycline treatment with 20 and 200 ng/mL, respectively, from one of the experiments performed in Fig. 5G, H. (**G**) RNA-level quantification of endogenous YTHDC1 upon 3′-UTR designed siRNA transfection. (**H**, **I**) Parental A549 cells treated with concentrations of Doxycycline applied for YTHDC1 inducible system. Data information: All data are shown are representative of at least three independent experiments. For (**C**, **D**, **G**), data are presented as mean ± s.d. ns, not significant $P > 0.05$, *$P \leq 0.05$, **$P \leq 0.01$, ***$P \leq 0.001$, paired two-tailed Student's *t* test was performed.

© The Author(s)
